# 3D printing for soft robotics – a review

**DOI:** 10.1080/14686996.2018.1431862

**Published:** 2018-03-08

**Authors:** Jahan Zeb Gul, Memoon Sajid, Muhammad Muqeet Rehman, Ghayas Uddin Siddiqui, Imran Shah, Kyung-Hwan Kim, Jae-Wook Lee, Kyung Hyun Choi

**Affiliations:** a Department of Mechatronics Engineering, Jeju National University, Jeju, South Korea; b Faculty of Electrical Engineering, Ghulam Ishaq Khan Institute of Engineering and Technology, Topi, Pakistan

**Keywords:** 3D Printing, soft robots, functional materials, biomimetic, 60 New topics/Others, 211 Scaffold / Tissue engineering / Drug delivery

## Abstract

Soft robots have received an increasing attention due to their advantages of high flexibility and safety for human operators but the fabrication is a challenge. Recently, 3D printing has been used as a key technology to fabricate soft robots because of high quality and printing multiple materials at the same time. Functional soft materials are particularly well suited for soft robotics due to a wide range of stimulants and sensitive demonstration of large deformations, high motion complexities and varied multi-functionalities. This review comprises a detailed survey of 3D printing in soft robotics. The development of key 3D printing technologies and new materials along with composites for soft robotic applications is investigated. A brief summary of 3D-printed soft devices suitable for medical to industrial applications is also included. The growing research on both 3D printing and soft robotics needs a summary of the major reported studies and the authors believe that this review article serves the purpose.

## Graphical abstract

**Figure:**
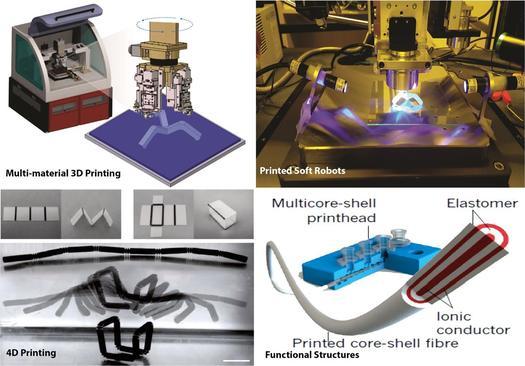

## 1. Introduction

3D printing is an additive manufacturing (AM) process defined as ‘the process of joining materials to make objects from 3D model data, usually layer upon layer, as opposed to subtractive manufacturing methodologies, such as traditional machining’ [1,2]. 3D printing can deliver parts of very sophisticated and complex geometries with no need of post-processing, built from custom-made materials and composites with near-zero material waste, while being applicable to a diversity of materials, including smart materials such as shape memory polymers and other stimulus-responsive materials. Therefore, 3D printing is a technology that offers increased ‘design freedom’ and allows designers and engineers to create unique products that can be manufactured at low volumes in a cost-effective way. One of the main example of the design freedom offered is that conventional assemblies can be restructured in a single complex structure that could not be manufactured with the current manufacturing processes. Another driver of the 3D printing technology is that it is environmentally and ecologically favourable. 3D printing technologies and methods are growing frequently in terms of application and market share, spreading into various manufacturing divisions, such as robotics, motorized, health and aerospace and are expected that this substantial growth will continue over the next few years.

In the last few years, there has been a significant trend towards the use of 3D printing technology to fabricate soft robots for various applications. Soft robots is a very young research area and mostly inspired by nature mechanisms which are optimized since centuries for a particular task. Mechanical robots and machines are made of hard materials that limit their ability to elastically deform and adapt their shape to external constraints and obstacles. Although they have the capability to be extremely powerful and precise, these rigid robots tend to be highly specialized and rarely exhibit the rich multi-functionality of nature. The soft robots are the next generation of robots which are elastically soft and capable of safely cooperating with humans or steering through constrained environments. Just as a mouse or octopus can squeeze through a small hole, a soft robot must be elastically deformable and capable of steering through narrowed spaces without inducing damaging internal pressures and stress concentrations [3,4].

The soft robots are primarily composed of fluids, gels, functional polymers and other easily deformable matter. These materials exhibit many of the same elastic and rheological properties of soft biological matter and allow the robot to remain operational even as it is stretched and squeezed. More importantly, all these materials are compatible with the current 3D printing technology. Conventional soft robot fabrication approach involves moulding, and casting is increasingly replaced with 3D printing technology because 3D printing is faster and more reliable. While a number of comprehensive reviews exist that focus on individual 3D printing technologies [2,5–26], 3D printable materials [12,27–29], soft materials [30,31], soft actuators [32–34] or specific applications [35–41] exist, a review of 3D printing in soft robotics is still absent. This review comprises a detailed survey of ongoing 3D printing techniques for soft robots. In an effort not to overwhelm the reader, the scope of the paper is limited in several ways. The focus is on 3D printing fabrication technologies with soft structure examples, materials that can be 3D printed for soft robotic applications and soft medical devices that are 3D printed. The review is divided into four sections. In Section 1, we overview the 3D printing technologies for soft robots, Section 2 is related to printable smart materials, Section 3 focuses on biological 3D-printed soft robots for *in vivo* and *in vitro* studies and we close with a broader perspective recommending future research directions and applications.

### 1.1. Relation between 3D printing & soft robots

Soft robots do not require fluidics, pneumatics or inflation instead of which they need tendons, shape memory coils, muscle-like actuators, etc. [42]. Hence, they can be built from commercially available soft materials and 3D printers, with a drawback that such materials cannot be transformed in every desired way. Furthermore, 3D printing has a limitation of speed and difficult scalability so currently the work on soft robotics is going on within the technological constraints of currently available 3D printers. 3D printing is a very slow process, but this is not a major issue, as no-one at this initial stage is looking for the mass production of soft robots. Yet the high specificity and ability to print the most complex shapes makes 3D printing an extremely attractive choice for the fabrication of soft robots. Power sources are an integral part in most of the newly developed soft robots [43] and 3D printing is an extremely useful technique to intelligently place them inside soft structures. However, one fundamental concern in using 3D printing technology for developing soft robots is that 3D printable soft materials have a large tendency to deform under the normally used forces during the building process due to their own weight so a support material becomes a necessity. There is room for the development of such a material that can become soft after being printed as a rigid structure. There are commercially available 3D printers with the ability to develop complex structures through viscoelastic hydrogels to be printed in span with self-supporting structures [44]. Hydrogels can bear pressures from kilopascals to megapascals. The range of such materials has recently been extended to other soft materials and elastomers. The research group of Jennifer A. Lewis working on the applications of 3D printing technology has developed an omnidirectional technique with the ability to print extremely soft materials such as liquids that can be held in place through polymerization at a later stage [4]. The problem of providing a support material is well addressed while using the technique of photolithography for the development of soft structures as printing takes place inside a resin bath in which the unused resin can restrict the deflection of soft materials. A research team based on the students of Delft University of Technology has introduced an additional feature for 3D printing that has the ability to cast silicones in a 3D-printed shell [45]. This low cost and advanced technique can be used to achieve new heights by creating those soft robotic products that were not possible earlier. This technique is known as UltiCast; it can print extremely complex shapes that are very difficult to achieve through typical 3D printing techniques such as fused deposition modelling (FDM) because each hot filament layer deforms the subsequent filament layers. A soft actuator can be printed inside a mould through the technique of UltiCast as it will eradicate the manual casting process hence resulting in faster speed. The freedom to personalize the robotic behaviour through controlling the robot geometry has allowed to print a custom-made soft gripper. Low cost 3D printing process can have several applications in soft robotics. It has another inspiring aspect that it can be extremely useful in the medical sector as it can reduce the cost of operation with assisted movement. A soft robotic glove with soft actuators inside it was recently developed through 3D printing and can be helpful in moving human fingers. This soft robotic 3D-printed glove can be useful for those who are suffering from limited hand function, local paralysis and arthritis or it can be used as a rehabilitation tool. Shape morphing materials with photosensitivity, thermal activation and responsive to water can also be printed now a days through 3D printing technology. These materials are extremely useful for soft robotic applications as they can be brought to any desired shape using heat, light or water. The scientists have even fabricated a 3D-printed cat tongue as a development in soft robotics after the successful experiments of a jamming-based gripper, a prosthetic hand and a 3D-printed soft robot with four legs that can walk on non-uniform and rough surfaces such as pebbles and sand. Such soft robots can take part in rescue operations or used for the applications of collecting sensor data from dangerous environments. This could only become possible due to the ability of 3D printing technology to build extremely complex structures consisting of both soft and hard materials inside soft robot legs. The ability of integrating soft and hard materials in a single structure can lead us to the realization of more compliant next generation soft robots that will be safer for human contact than their predecessors. Researchers have also managed to develop a soft robotic hand through 3D printing technology with the ability feel surfaces like the natural hand of human beings. Unlike other soft robotic hands that grip and sense through motors, this newly developed hand by Shepherd and his team uses its external fingertips to collect data through actually feeling the responsiveness internally [46]. Such advancements in the field of soft robotics were not possible had it not been the technological progress of 3D printing technology over the last two decades.

### 1.2. 3D-printed biomimetic soft structures

**Figure 1.**
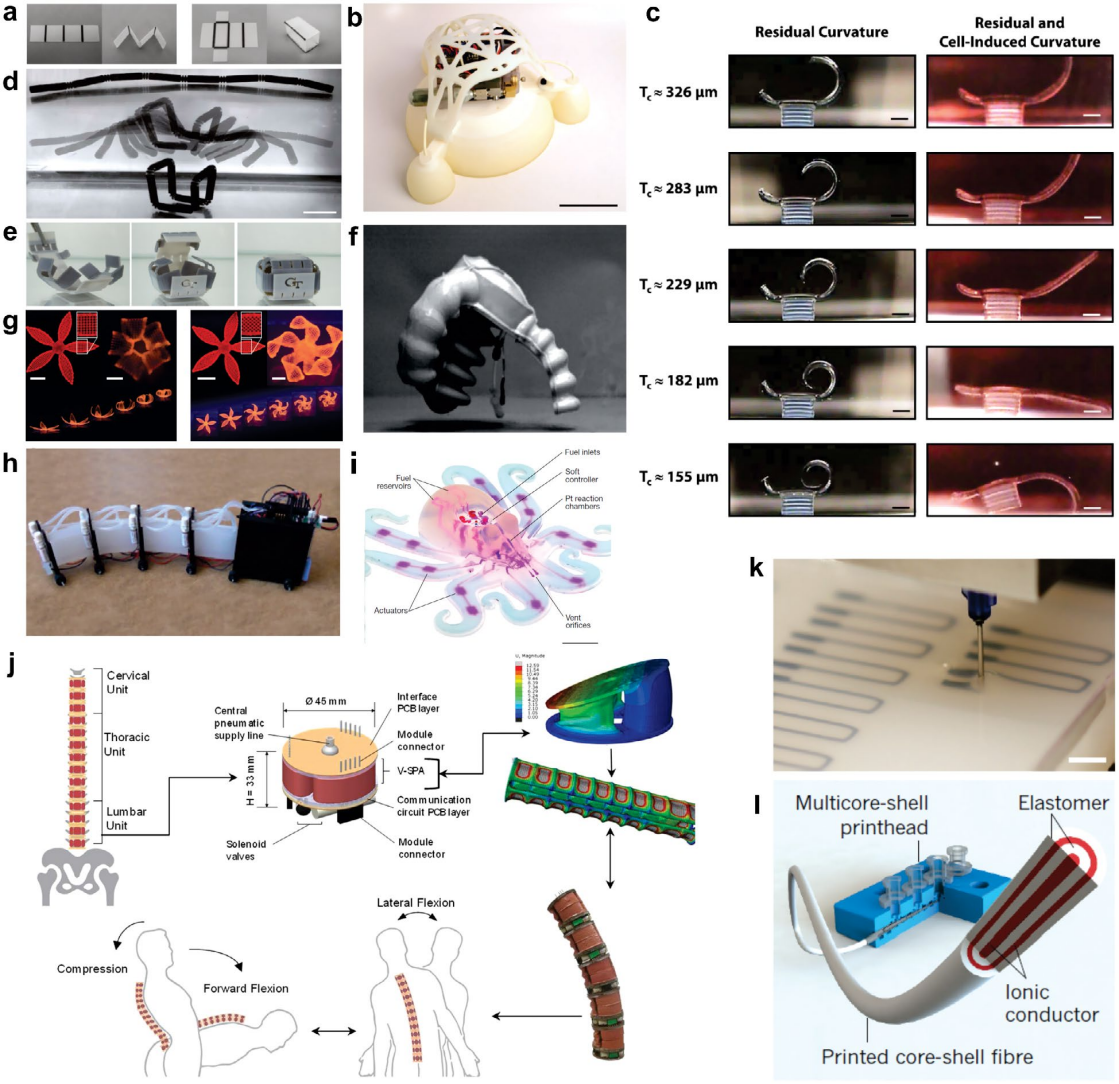
Examples of printed soft robots and soft devices. (a) Pre-strained polystyrene substrate with inkjet-printed hinges made of carbon black ink. (b) 3D-printed jumping soft robot. (c) 3D stereolithography-printed bat with curvature time lapse. (d) 4D-printed composite with swellableable hinges. (e) 4D-printed unfolded box composed of shape memory polymers. (f) A jumping soft robot with 3D-printed mould. (g) 4D printing of hydrogel composites for soft robotic applications. (h) A snake inspired soft robot with 3D-printed mould. (i) Multi-step 3D-printed octobot. (j) Pneumatic actuator for spinal compression and flextion with 3D-printed mould. (k) Embedded 3D printing of soft strain sensor for soft robots. (l) Multicore print head shell capacitive sensor.

3D printing technology gives much freedom to designers and also simplifies the execution of effective robotic design principles, such as separation of control and power actuators. It also enables the investigation of mechanically complex designs. Wehner et al. reported the fabrication of an integrated design strategy for an entirely soft and autonomous robot inspired by the octopus. They used a hybrid fabrication technology including moulding, soft lithography and multi-material embedded 3D printing. The rapid fabrication approach and integrated design proposed in this study facilitates the programmable assembly of several materials within a single body to realize an entirely soft robot [47]. Mosadegh et al. developed a pneumatic network for the rapid actuation of soft robots fabricated through hybrid technique of 3D printing and lithography using elastomeric materials. A new design of pneumatic networks was proposed in this study. The advantage of their design is the reduction in the required level of gas needed for inflation of such networks resulting in much faster actuation. The fabricated actuators can be operated for more than a million cycles without any noticeable degradation in the obtained results [48]. Martinez et al. designed and fabricated the soft tentacles using the 3D printing technology of additive manufacturing for the fabrication of moulds for elastomeric casting. These soft tentacles were based on micro-pneumatic networks spatially distributed at the interface of two different elastomers with the ability of complex 3D motion. The range of motion and the ability of these tentacles to grip different objects with arbitrary shapes was successfully demonstrated in this work [49]. Song et al. reported the use of soft pneumatic actuators for the purpose of providing a physical support that would help in healing the spinal cord injuries of penalized animals by providing support to hip joint movement. The experiments were carried out on a living rodent. To perform the required study, a soft robot was fabricated through 3D printing technology. The body of the soft robot consisted of three main parts including mainframe, soft actuators and the soft couplings [50]. Umedachi et al. fabricated a 3D-printed soft robot to mimic the motion of a caterpillar. They termed it as a 3D-printed soft (3D-PS). This 3D-PS has the ability to crawl, inch and steer like a real caterpillar. This motion was made possible by embedding a combination of shape memory alloy (SMA) wires in the body of 3D-PS and by passing electric current through them. The movement of 3D-PS was restricted to forward and backward direction only. The fabrication of these 3D-PS robots using 3D printing approach was quick, simple and cost-effective [51]. Kim et al. fabricated soft skin module using 3D-printed additive manufacturing for the application of safe interaction between a human and robot. Entire fabrication of module prototypes was carried out using a multi-material 3D printer. This soft skin module strongly diminishes the effect of impact forces due to collisions. These modules can be attached to various robotic systems with the ability of very gentle physical interaction with soft objects [52]. Bartlett et al. used multi-material 3D-printing technology for the manufacturing of a combustion-powered robot. This robot gained power from the combustion reaction between oxygen and butane to perform autonomous jumping. This approach encouraged high-throughput prototyping by allowing quick design repetition with no added cost for increased morphological difficulty [53]. Katzschmann et al. presented the fabrication of a hydraulic autonomous soft robotic fish and illustrated its locomotion in three dimensions. They used 3D printing technology for the fabrication of soft body parts to develop robotic fish allowing arbitrary internal fluidic channels and a wide range of constant body deformations for continuous bending. The nose of the fish was also fabricated through 3D printing that acted as a waterproof house for the installed electronics such as motor driver, microcontroller and wireless communication system [54]. Onal and Rus fabricated a soft robot through 3D printing technology that was bio-inspired from the shape and motion of a snake with the ability to undulate in a similar pattern to a real snake using the actuation power without human assistance. The as-fabricated soft snake robot was autonomous with onboard computation, control, power and actuation capabilities. The robot had four bidirectional actuators to create a wave through its entire body from head to tail. The time it took to fabricate the soft robotic snake was 14 h with the ability to achieve an average locomotion speed of 19 mm s^−1^ [55]. Homberg et al. applied the technique of 3D printing for the fabrication of a soft robotic hand with multi-fingers and ability to grip various solid objects such as a CD, paper, pen, soda can etc. Resistive bend sensors were installed in each finger to distinguish between different objects. It had the ability to recognize a set of objects owing to the stored data from internal flex sensors. Each finger of the soft robotic hand had an independent sensing ability [56]. Umedachi et al. fabricated a soft worm robot by 3D printing technology, having high deformable capability from rubber like material. The reported soft worm does not require any fluidic or pneumatic actuators as they are powered electrically through SMA coils. The results of this study had important inferences for the ongoing research on soft animal locomotion and for designing other multipurpose deformable robots [57]. Figure 1 shows examples of 3D-printed soft robots for various applications.

## 2. 3D printing technologies

**Figure 2.**
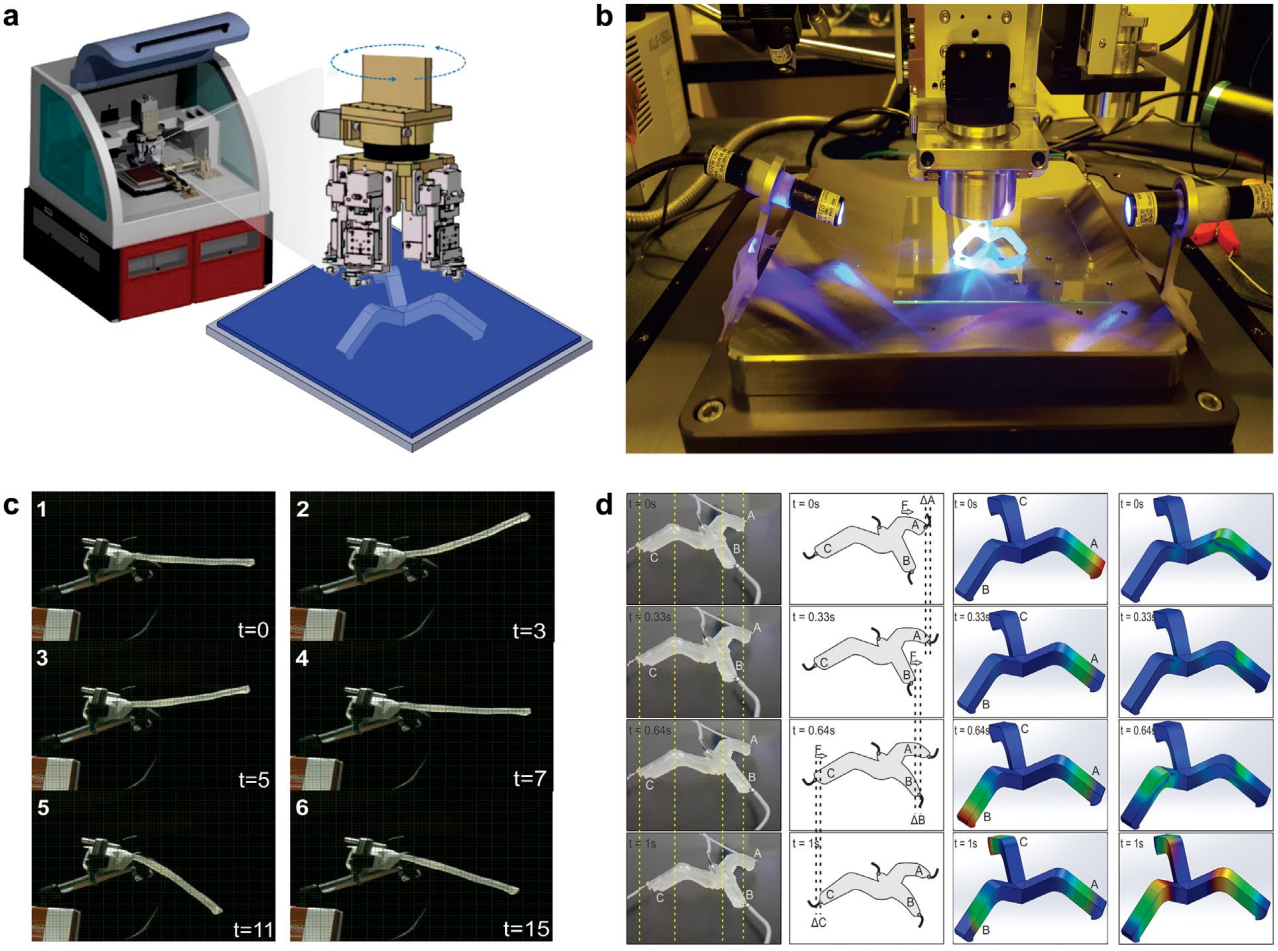
(a) Multi-material 3D printing system by Advanced Micro Mechatronics (AMM) Research Lab, Jeju National University, South Korea. (b) Photograph of the AMM’s multi-material 3D printing system. (c) Soft omnidirectional actuator by AMM Lab. (d) (i) Fabricated soft-bot actuation of each leg at different time intervals. (ii) Model of actuation to generate movement at different time intervals. (iii) Finite-element displacement simulation results of one complete actuation cycle. (iv) Finite element strain simulation of one complete actuation cycle.

**Figure 3.**
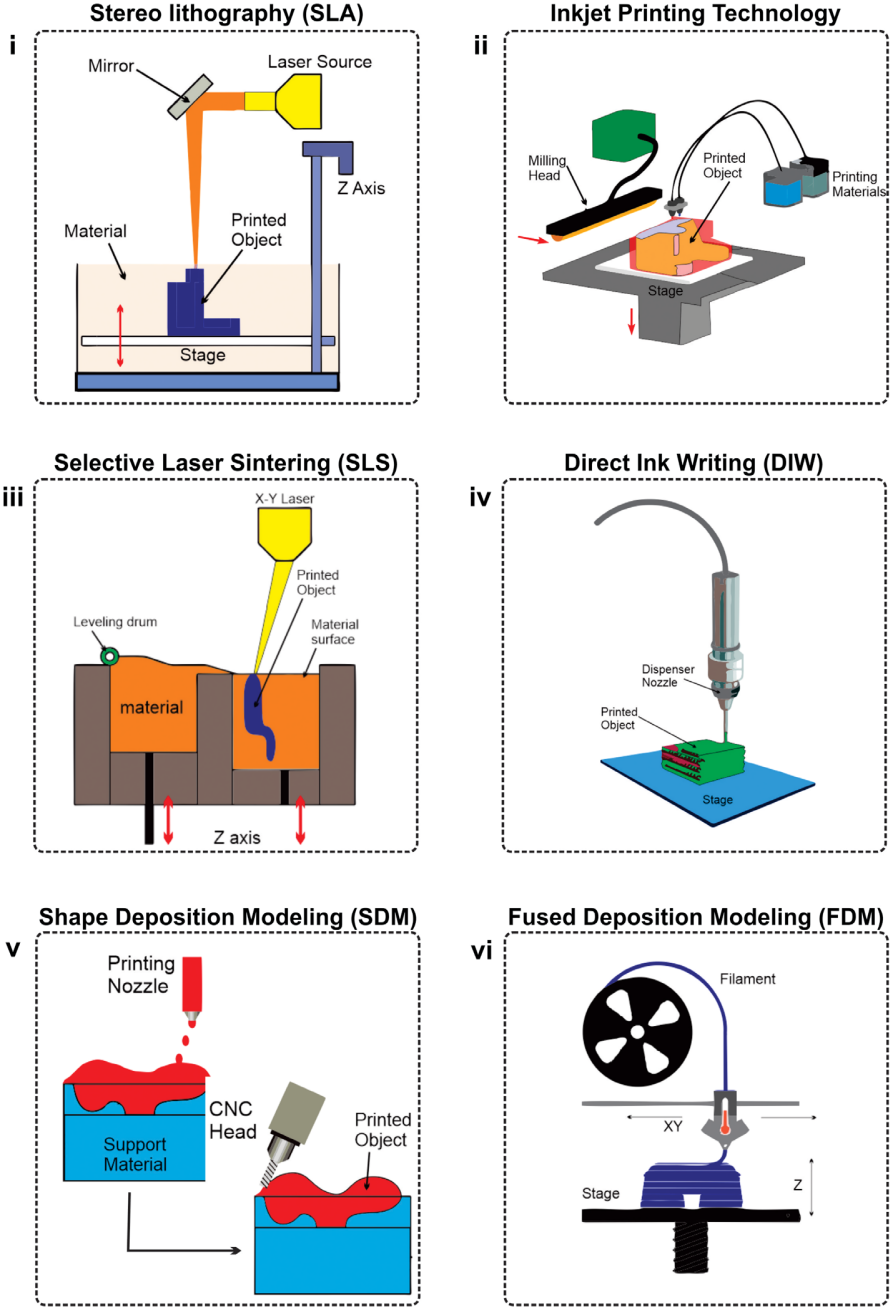
3D Printing techniques used to fabricate soft robots. (i) A liquid resin is selectively photo-polymerized in the process of SLA by a laser. (ii) Inkjet printing is similar to SLA in many ways with a difference that a movable inkjet head is used in this technique to apply a photopolymer being activated by a UV lamp. (iii) The powder of metal material is rolled across a build platform and a laser is directed into the powder followed by rolling the powder over the top of as-deposited layer and this process keeps on repeating till the desired 3D object is completely fabricated. (iv) DIW is an alternative printing technique to FDM for additive manufacturing of desired objects under ambient conditions in which ink passes through a nozzle in a controlled manner. (v) Shape deposition modelling technology consists of several steps including deposition. The material in heterogeneous deposition is changed between each deposition process. (vi) Soft materials are printed in the form of a continuous filament in FDM method with a single layer being deposited at a time.

In this section, we will present introduction of diverse fabrication techniques based on additive manufacturing to show its ability to produce simple and complex soft robots with various applications. In additive manufacturing, a computer-controlled transformation stage typically changes a pattern-generating device, either in the form of an ink-based print head or laser optics to fabricate the desired objects in a layer by layer pattern. During the process of additive manufacturing, patterned regions composed of powders, inks or resins are solidified to produce the desired 3D shapes. These 3D-printed objects are a physical realization of the digital designs. Several basic additive manufacturing printing techniques have been presented since the introduction of 3D printing. The technology has advanced from making basic prototypes to fabricating finished products. The explicit solidification and patterning method used by a given additive manufacturing technique exhibits the minimum feature size that can be achieved and the sort of printable soft materials that can be used. The major differences in the available basic printing methods are mainly related to increasing printing speed, enhancing printing resolution, reduced material consumption and deploying multiple materials in the printing of a desired 3D object [58]. The role of these basic additive manufacturing techniques in the field of soft robotics is presented below. Figure 1 shows the multi-material 3D printing system by Advanced Micro Mechatronics (AMM) Research Lab, Jeju National University, South Korea and few printed soft robots (Figure 2). Table 1 summarizes the 3D printing technologies with respect to soft robots. Working principles of six main 3D printing technologies used to fabricate soft robots are illustrated in Figure 3.

**Table 1.** Summary of 3D printing technology with respect to soft robots.

Mechanism	Robot part	State of starting material	Layer creation technique	Materials	Size	Advantage	Disadvantage	Application	Sub-Heading number	Reference
Stereolithography (SLA)	Bio-bot arrays		Layer by layer	Poly(ethylene glycol)		Less resin material, large build volume, Precise control, Rapid polymerization	Post-curing, warping, brittle parts with a tacky surface, support required, little choice of material, unused material is toxic	Miniaturized Walking	I	[59]
				Diacrylate (PEGDA)						
								Biological Machines		
	Multi-material cantilevers		Layer by layer	Poly(ethylene glycol) diacrylate (PEGDA) and acrylic-PEG-collagen (PC) mixtures	2 × 2 × 4 mm					[60]
	Actuator		Bottom up approach	Elastomeric precursor; Spot-E resin, Spot-A	40.0 × 71.1 mm^2^			Octopus Tentacles		[61]
Inkjet Printing	Bellows actuators, gear pumps, soft grippers and a hexapod Robot,	Liquid	Multi-material layer by layer printing	Tangoblack+	14 × 9 × 7 cm			Hydraulically actuated robots	Ii	[62]
	Complete robot		Multi-material UV-curable printing	Urethane and epoxy	80 × 5 × 5 mm			Tri-legged soft bot with spider Mimicry		[63]
	Mould			ABS and silicon rubber				Caterpillar-inspired soft-bodied rolling robot		[64]
Selective laser sintering (SLS)	Bellow actuators		Top-Down	Elastic silicone material		No support required		Soft robotic hand	Iii	[65]
	Flexure hinges			Polyamide (PA 12, Nylon)	0.1 mm × 0.5 mm			Soft Robot Kinematics of snake		[66]
DIW	Extensible sensing skin			Silicone elastomer, hydrogel elastomers, polyacrylamide etc.	3.75 × 3.75 cm		Unable to fabricate continuous fibre-reinforced composites	Tactile Machines with kinesthetic sense	Iv	[69]
SDM	Cockroach Limbs			Viscoelastic polyurethane	120 mm × 120 mm × 50 mm			Biomimetic Components	V	[70]
	Small robot limbs			Viscoelastic polyurethane	–			Biomimetic Robotic Mechanisms		[71]
	Fingers of robot			Two-part industrial polyurethanes				Robust compliant grasper		[73]
	Hexapedal Robots			Viscoelastic polyurethane, polyester fibres and low melting temperature wax	16 cm			Performance and locomotion Dynamics of insects		[72]
	Fingers of Grasper			Polyurethane elastomer	116 cm^3^			Soft, Atraumatic and Deployable surgical grasper		[74]
Fused deposition modelling (FDM)	Actuator		Layer by layer	Silicone elastomer	50 × 20 × 40 mm			Soft robot prototypes	Vi	[75]
	3D structures		Layer by layer	Nafion	5 mm × 10 mm × 0.5 mm			Macro-scale soft robotic systems		[76]
	Actuator modules			Silicon rubber elastomer	–			Soft snake		[77]
	Flexible Fingers			Poly(vinyl chloride) (PVC) sheets	–			Soft prosthetic Finger		[78]
	Soft pneumatic actuators			Thermoplastic elastomer filament ninjaflex (ninjatek, PA)	150 mm × 25 mm × 11 mm			Soft Robotic applications		[79]
3D Printing				Fugitive (Pluronic F127) and catalytic inks				Entirely soft octobot with embedded electronics		[47]
	Moulds			Elastomeric silicon				Pneumatic networks for soft robotics		[48]
	Moulds			ABS	15 cm			Robotic Tentacles		[49]
	Soft Actuators, main frame		Layer by layer	ABS Plastic	30 × 10 mm			Rehabilitation of spinalized rodents		[50]
	Whole body		Multi-material printing	Tangoplus and veroclear	8 cm			Mimicking of caterpillar motion		[51]
	Soft Skin		Multi-material printing	Tangoplus	173 cm^3^			Safe human-robot interaction		[52]
	Robot Body		Multi-material printing					Combustion-powered robot		[53]
	Outer mould, lid, model core		Layer by layer	Silicon rubber	0.45 m × 0.19 m × 0.13 m			Hydraulic autonomous soft robotic fish		[54]
	Mould, passive wheel, valve holders, tail enclosure			Silicon rubber				Dynamics of a fluidic soft robot		[55]
	Mould			Silicon rubber	2.5 × 2.5 × 11 cm			Soft Robotic Gripper		[56]
	Softworms			Deformable rubber-like Polymer				Bio-inspiration soft robots		[57]

### 2.1. Stereo lithography

A liquid resin is selectively photopolymerized in the SLA process by a laser. After the printing first layer, a new layer is introduced and afterward cross-linked by local illumination. This process of depositing liquid resin layer by layer is repeated several times until the printing of chosen 3D object is finished. Various other novel additive manufacturing techniques such as continuous liquid interface production (CLIP), digital projection lithography (DLP) and two-photon polymerization (2PP), works on the same concept of SLA printing. However, one basic difference between SLA and these techniques is that SLA depends on point-source illumination in order to pattern one part of a single layer at a time whereas CLIP and DLP have the ability to solidify a complete layer using dynamic liquid-crystal masks for the projection of a mask pattern. There is a trade-off between several parameters of additive manufacturing such as build volume, printer resolution and printing speed. DLP and CLIP are superior to SLA in printing speed whereas 2PP has the highest lateral resolution (~100 nm) of 3D-printed parts owing to the benefit of the squared point-spread function. Highest resolution of 2PP makes it an ideal choice for the fabrication of extremely complex micro-architectures with the limitation of reduced volume (~1 cm^3^) as compared to CLIP that can print much larger volumes (~100 cm^3^) with the limitation of lower printing resolution. SLA and other novel additive manufacturing techniques (CLIP, DLP and 2PP) based on its basic concept have the limitation that multi-materials cannot be printed in a single go by either of these techniques [58]. Chan et al. fabricated a locomotive bio-bot using the additive manufacturing technique of stereolithography based on cardiomyocytes and hydrogels. Biological bimorph structure was used as the actuator for powering the bio-bot. Locomotive motion of various designs of bio-bots was tested by changing the thickness of cantilever. The extreme recorded speed of the bio bot was 236 m s^−1^ with an average displacement of ~354 m at a beating frequency of ~1.5 Hz [59]. In another work, Chan et al. fabricated a multi-material hydrogel actuators and cantilevers with an elasticity up to 10^3^ kPa using the stereolithography technique. The use of SLA allowed simple, quick and easy shifting of material using a single structure of 3D printer. Stress was created on the cantilevers through the traction forces created by the cardiomyocytes. These cantilevers can be used for the prototyping of cell-based bio-hybrid actuators [60]. Peele et al. fabricated and tested incompatible pairs of folded soft actuators with four degree of freedom inspired by the movement of octopus tentacles. These fabricated soft actuators had the ability to sweep through their entire range within 70 ms with intricate internal architectures. Soft structures were built using the additive manufacturing technique of stereolithography. The developed soft actuators had large actuation amplitudes. The proposed system is highly suitable for the development of soft machines that could interact and mimic the biological systems [61].

### 2.2. Photo-curable inkjet printing

Inkjet printing is similar to SLA in many ways with a difference that a movable inkjet head is used in this technique to apply a photopolymer being activated by a UV lamp. The liquid photopolymer is printed on a build platform and the deposited layers are activated by UV lights followed by printing of additional layers in a similar manner. Both additive manufacturing printing techniques of SLA and SLS are light-based methods that are dependent upon laser. Although these techniques have superior feature resolution but they have the limitation of yielding rigid thermoset polymers by patterning with either only thermoplastic polymer powders or photopolymerizable resins. Comparatively the second classification of printing techniques i.e. ink-based additive manufacturing methods have the added advantage of printing patterns using numerous soft materials in the form of printable and formulated inks using a wide variety of molecular, particulate or polymeric species. Such ink-based additive manufacturing techniques can be categorized on the basis of various parameters such as ink’s viscosity, shear yield stress, loss moduli, surface tension and shear elastic.

Several additive manufacturing methods use the concept of droplet-based printing techniques such as inkjet printing on a powder bed, direct inkjet printing and hot melt printing. Soft materials deposited in droplet-based printing are similar to those of 2D forms. The inks used in these printing techniques have low viscosity. The drop formation in these ink-based printing methods is highly dependent upon the printing parameters and characteristics of ink to be printed. These characteristics of used ink include ink’s viscosity (*μ*), ink’s density (*ρ*), diameter of droplet (*L*), surface tension (*γ*), nozzle diameter (*d*) and velocity of ejected droplet (*v*). For the successful printing of desired objects through inkjet printing, all these mentioned characteristics must be precisely controlled in order to attain the right tradeoff between inertial forces, viscosity and surface tension. The fluid dynamics involved in drop wetting, formation and spreading play a limited but vital role in defining the resolution and surface roughness of the desired objects. Typical values for a few of the most important characteristics of inks are *L* (10–30 μm), *μ* (2–20 mPa s) and *v* (1–10 ms^−1^) respectively. The high dependence of printing parameters through ink-based techniques imply that it is difficult to avoid clogging of nozzle during jetting ink of complex materials such as polymer inks with high concentration. However, this disadvantage is neutralized by the several other advantages offered by these printing methods including huge variety of printable materials, their well-established multi-nozzle arrays with the ability of supplying 100 million drops/s and their sophisticated designs of print heads [58]. MacCurdy et al. fabricated a hydraulically actuated hexapod soft robot in a single step using bellows actuators, soft grippers and gear pumps through multi-material 3D-inkjet printing system. They proposed a new process of inkjet printing for the simultaneous fabrication of desired 3D object using liquid and solid components and called it as printable hydraulics with the ability to carry out complete fabrication with diverse functionality of hydraulically actuated soft robotic structures. Furthermore, the applications of such soft robots fabricated through additive manufacturing have also been demonstrated in this work [62]. In our previous work, we fabricated an *in situ* UV curable multi-material tri-legged soft bot inspired by the multi-step dynamic forward movement of a spider. A commercially available bio metal filament was used as an actuator embedded into the soft legs of a soft bot. The 3D printer used for the fabrication of this tri-pedal soft bot was custom made with a rotational multi-head inkjet printing system along with various lasers of different wavelengths. The whole fabrication was carried out using two soft materials such as polyurethane and epoxy in three-layered steps. The fabricated soft bot has the ability to move in a forward direction with a speed as high as 2.7 mm/s at a frequency of 5 Hz when applied with an input voltage signal of 3 V and a current equal 0.25 A respectively [63]. Lin et al. fabricated soft-bodied rolling robot in which they mimicked the motion of a caterpillar that can generate their own momentum by curling themselves in the form of a round shaped wheel to escape from danger situations at a high speed of 0.2 m s^−1^ within 100 ms. The as-fabricated soft bot was helpful in exploring the control issues and dynamics of surface-to-air rolling. This caterpillar soft bot also provided an estimate of the mechanical power required for rolling that was close to a locust jump [64].

### 2.3. Selective laser sintering

Primarily metals are used as the deposition material in additive manufacturing through SLS to form desired 3D objects but post-processing such as sintering, infiltration and finishing is required for completing device fabrication. The powder of metal material is rolled across a build platform and a laser is directed into the powder followed by rolling the powder over the top of as-deposited layer and this process keeps on repeating till the desired 3D object is completely fabricated. The unused powder that does not form into the fabricated part of the 3D object remains in the build platform to support the object. Apart from metals, polymers are also used in SLS. Local treatment of polymer particles is carried out in a powder bed through heat by fusing them together with the help of rastering laser. Local sintering of the subsequent powder layer is carried out after printing a layer across the bed. Granulated powders with a typical diameter in the range of 10 –100 μm are used to facilitate the spreading. During the building process of the desired 3D object, the non-fused sections in the powder bed play the role of support material. In order to reduce material consumption, the unattached powder is detached and reused after completing the fabrication of chosen 3D model and removing it from the powder bed. The minimum achievable pattern size using SLS is ~100 μm, slightly larger than the average particle size available in the powder bed [58]. Rost and Schadle developed a multi-finger soft robotic (4 fingers) hand with 12 degree of freedom using SLS additive manufacturing technique. This hand mimicked a human hand and consisted of 12 pneumatic bellow actuators. This robotic hand had the ability to perform complex functions such as desired lifting, gripping, spinning and precise positioning of an object [65]. Roppenecker et al. used PA 2200 based on polyamide (PA 12, Nylon) material to fabricate multi-arm snake-like robot by SLS fabrication technique. They build various soft structures based on flexure hinges such as cup spring structure and helical structure that can be helpful in performing surgery inside the stomach tract. These SLS made structures had the ability to carry ~800 g of weight [66].

### 2.4. Direct ink writing

DIW is an alternative printing technique to FDM for additive manufacturing of desired objects under ambient conditions in which ink passes through a nozzle in a controlled manner. It is layer by layer addition technique in which materials are added both in planer and 3D form. The ink selection depends on its flowing parameters such as viscosity, surface tension, shear stress and shear elastic modulus. Currently, the DIW technique offers the widest spectrum of printable materials such as electrical, biological and structural materials [58]. Different ink materials used are a colloidal suspension, hydrogels, thermoset polymers and fugitive inks [67]. The distinguishable advantage of DIW printing method is its ability to print fugitive organic, filled epoxy inks and concentrated polymers with the fluid properties essential for the deposition of complex 3D designs. Characteristic values for the various ink parameters for DIW technique include printing speed (1 mm s^−1^ to 10 cm s^−1^), ink viscosity (10^2^–10^6^ mPa s) and minimum filament diameter (1–250 μm) respectively. The magnitude of yield stress must be greater than the applied stress in the print head in order to induce flow through the printing nozzle. Additional processing steps like thermal curing or photopolymerization might be required in some cases to completely solidify the desired printed objects. Avoiding such additional steps from FDM printing process might result in the undesired finishing of desired 3D objects as the subsequent printed layers might not be well supported by the previously printed layer. Such a deficiency can be overcome by coupling print heads with hot chambers or ultraviolet light emitting diodes.

Ink-based printing techniques such as inkjet printing, DIW and FDM can simply be used for the additive manufacturing of multi-materials. DIW offers the broadest range of printable materials, including biological, electrical and structural materials. Multi-material DIW can be realized either using microfluidic print heads with the flexibility of switching, core-shell printing and mixing or using multiple (single-nozzle) printheads, each with the different ink formulation. These microfluidic switching nozzles have the ability to swap between two different inks when desired. On contrary, mixing nozzles can be used to print materials with tunable conductive and mechanical properties of materials. Core-shell print heads produce filaments with concentrically layered materials with the added advantage of the dramatic increase in printing speed. Two different inks can be patterned simultaneously using double multi-nozzle arrays but the nozzle size of these multi-nozzle arrays are comparatively larger (100–200 μm in diameter) with another limitation that such nozzles cannot be addressed individually. Furthermore, a lot of efforts are being made recently in directly writing inks by embedded 3D printing with the ability to fabricate desired objects of soft materials. These options of DIW additive manufacturing technique offer a substantial flexibility in the design and forms of 3D printable shapes [58]. Complex 3D shapes can be designed and fabricated rapidly using DIW printing technique. It does not require dies, expensive tooling or lithographic masks. DIW offers cost reduction, wide variety of materials and fabrications of arbitrary 3D structures that are essential for advancement in multidisciplinary research [68].

Robinson et al. demonstrated an artificial equivalent of sensory-motor onto soft, fluidic elastomer actuators (FEAs) through DIW printing technique. They used two inks for their study including electrically insulating silicone and an ionically conductive hydrogel. Sensors were fabricated to allow tangible sensing and kinesthetic response in a pneumatically stimulated haptic device. The as reported capacitive skin allowed the detection of a compressive force of ∼2 N generated through pressing a finger on its top surface with an internal pressure of ∼10 kPa [69].

### 2.5. Shape deposition modelling

Shape deposition modelling technology is used for fabrication of complex geometries with heterogeneous materials mainly for the application of rapid prototyping. This is a cyclic process that consists of several steps including a deposition. The material in heterogeneous deposition is changed between each deposition process. Although this method was much better than traditional manufacturing processes used for fabrication; however, there were some certain limitations in using this methodology as well such as high control is required, proper bonding among materials and imperfect machining of plastic leads to fatigue failure due to imperfection of surfaces. Xu et al. have reported the fabrication of cockroach limbs for the first time using a soft, viscoelastic polyurethane material fabricated through SDM technology. They studied the damping and stiffness of these legs and their obtained model results were inspiring at low frequencies; however, they were not that appealing at higher frequencies. This study can inspire researchers to develop a novel material to further enhance the viscoelastic movement of the cockroach leg in a wide frequency spectrum [70]. Bailey et al. fabricated a five-bar mechanism in which joints are replaced by flexures. They discussed the fabrication and design of small robot limbs through the additive manufacturing technology of SDM with locally embedded actuators and sensors and changing stiffness. The material used for bar linkages was polyurethane owing to its high stiffness and soft viscoelastic nature. They studied the compliance and damping effects in this five-bar mechanism [71]. Cham et al. fabricated hexapedal robot by SDM. Their developed prototype was capable of attaining a maximum speed of 3.5 body length per second (55 cm s^−1^). The prototypes were robust and optimal in performance because design principles applied to these models were taken from biological studies of running animals [72].

Dollar et al. used the additive manufacturing technique of SDM to fabricate a complete robotic grasper with soft fingers with the functionality of typical metal prototypes with negligible complexity. The as-fabricated gripper was extremely robust while maintaining the benefits of joint compliance with the inherent properties such as robust construction, low passive contact forces and large grasp range. The grasper had the ability to move with a maximum and constant speed of 2 cm s^−1^ until it reaches and grasp the desired object successfully [73]. Gafford et al. recently reported another deployable surgical grasper fabricated through the same technology of SDM with pressure sensing board to grasp and manipulate some soft tissues during surgeries such as laparoscopic pancreatic surgery. The same group published another paper by improving their own design after feedback from surgeons in which they improved the surface interface of the tool with the tissues. Their design needed improvement in bending profiles of fingers in decoupling for more comfortability [74].

### 2.6. Fused deposition modelling

SDM is a solid freeform fabrication process which means it is built from start to finish rather than by removing excess materials from a given object. It does this by layering support material and the desired finished material. In this technology, support material is laid down as a base for the final material, final material is laid on top of the support material and computer numerical control (CNC) mills the part to the desired shape. Soft materials are printed in the form of a continuous filament in FDM method with a single layer being deposited at a time. 3D FDM provides the luxury of a wider range of printable geometries, their feature size and variety of ink designs. Thermoplastic filaments are fed by heating the extrusion head followed by solidifying them through cooling below their glass transition temperature in FDM. The famous printable plastic filament materials used in this method are polycarbonate, polylactic acid (PLA) and butadiene styrene (ABS). The polymer filaments offer a flexibility of being filled with carbon black particles in order to boost the functionality of the printed objects. FDM has become an extremely famous choice for additive manufacturing during the last decade owing to its compatibility with common materials and ease of use. FDM has the added advantage of being cost effective, user-friendly, highly reliable and requires very little post-processing. Both inkjet printers and FDM have the ability to print primary building materials beside sacrificial materials that back the spanning features. The drawbacks of FDM technology include low surface quality, low resolution and wrapping [58].

Morrow et al. fabricated soft robotic actuators for the application of building soft robot prototypes using the additive manufacturing technique of FDM. They observed that their printed soft actuators were able to perform like a moulded actuator with an average error of ~5% justifying its feasibility to be used in soft robotics applications. The internal and external diameter of the printed actuator by FDM was 15 mm and 20 mm respectively. It took them 40 min to complete the fabrication process [75]. Carrico et al. developed a fused filament-based soft active 3D structure using a composite of ionic polymer and metal. This group reported for the first time the sensing and actuation characteristics of the ionic polymer-metal composite by incorporating them into the 3D structural designs. The procedure of additive manufacturing adopted by this group has the ability to print micro- and macro-scale actuator and sensors for soft robotic systems. A precursor material was extruded into a thermoplastic filament followed by the use of this filament by a custom developed 3D printer to assemble the anticipated soft polymer structures. A chemical functionalization process was carried out to induce electro activity in the 3D-printed structures. The movement of these soft actuators was then controlled by applying external voltage supply through electrodes [76].

Onal and Rus developed a modular approach towards soft robotics by manufacturing a snake inspired soft robot fabricated through FDM technology of additive manufacturing. There work is based on the elastomeric actuation through mechanical energy transferred by the pressurized fluids. Such FEAs are cost-effective, safe to use, fast and highly adaptable to robotic systems. The velocity of locomotion of the as-fabricated soft snake robot was measured as 9.2 mm s^−1^. Each progressing step resulted in 52.3 mm of forward movement [77]. Mutlu et al. fabricated a completely compliant soft robotic human finger made of flexible poly(vinyl chloride)(PVC) sheets through FDM technology. The stiffness of the complete robotic finger was augmented mechanically by controlling the stiffness expanding unit. The obtained results illustrated that the finger stiffness could be increased up to 40% depending upon the used material and thickness of the expanding unit [78]. Yap et al. 3D-printed soft pneumatic actuators using thermoplastic elastomer filament NinjaFlex (NinjaTek, PA) through FDM technology. They claimed that FDM was preferred because other existing fabrication methods for realizing soft pneumatic actuators with complex geometries are either time consuming or require several steps. This group studied the material properties, collected simulation results for the mechanical performance of printed actuators and evaluated their bending ability, durability and mechanical strength with an illustration of possible soft robotic applications. They fabricated a soft gripper with the ability to grip and lift heavy entities with different shapes and size. They also developed wrist exoskeletons and wearable hand to illustrate complex movements like bidirectional bending of soft actuators [79].

## 3. 3D printable materials for soft robotics

Smart materials are the active materials that can undergo some observable change in one domain in response to external stimuli through another domain; the external stimuli may be thermal, chemical, mechanical, optical, moisture, pH, pneumatic and electric or magnetic field. Additive manufacturing or 3D printing of smart materials has been an astounding boost for researchers in the form of 4D printing and soft robotics. When smart materials fabricated by 3D printing in a particular shape have the potential to alter their given shape or properties with repect to time under the influence of some external stimuli, this phenomenon is called 4D printing [30]. Whereas, soft robotics is a broad term that includes actuators, artificial muscles, soft stretchable sensors, soft energy harvesting, pneumatic nets, electroactive polymers and soft electronics. The soft robotics is the field of mimicking of a natural organism using smart materials. This artificial organism paradigm has not only mimicked the shape and motion of some natural organism but now it is also going to exploit all the traits of a natural organism. The revolution in 3D printing has accelerated the progress in soft robotics; it involves two types of contributions: direct printing of smart materials, and 3D printing of moulds for soft robotics. We have spotlighted this review with both types of additive manufacturing in soft robotics. Smart materials which have been used in soft robotics or actuators for soft robotics are dielectric elastomer actuators (DEAs), hydrogels, electroactive polymers (EAPs), SMAs, shape memory polymers (SMPs) and FEAs.

### 3.1. Dielectric elastomers

3D printing of DEAs for soft robotics was first reported by Rossiter et al. [80]. DEAs are the class of EAPs that undergo a change in strain upon applying the electric field. DEA can be used as artificial muscle because it has the ability to mimic mammalian muscles due to its large strain, high energy density and light weight. In that work, authors presented the two-membrane antagonistic actuator having electrodes on both sides of each membrane. Upon applying the electric field to the upper membrane the actuator moved upward and vice versa.

In another work, DE was fabricated through 3D printing and a soft robot has been presented [81]. They 3D printed silicone films (Thickness ≈ 300 μm) and carbon grease films. The authors fabricated rectangular and circular DEAs, and through simulations and experiments they proved that rectangular DEA had larger actuation then circular.

### 3.2. Shape memory polymers

SMPs are thermally activated memory polymers that have a tendency to change their original shape when they are triggered by heat. SMP has been reported for actuation that was printed through 3D digital light processing printer [82]. Polycaprolactone (PCL) cross-linked by methacrylate was used as SMP; the printed structure of SMP was rigid at room temperature, having wax-like surface; when heated above the *T*_*m*_ (55 °C), it becomes pliant and elastomeric. At this state, any deformation can be fixated by cooling below *T*_*m*_ whereas by heating again the structure regains its original shape. The 3D printing of SMP was also reported through FDM technique [83]. SMP based on thermoplastic polyurethane elastomer (TPU) family was used in this study that was processed into filament to make it compatible for FDM to fabricate SMPs can lead to the fabrication minimal invasive medical devices, sensors, wearable electronics and soft robotics.

### 3.3. Hydrogels

3D printing of hydrogels have been studied for applications spanning from medical devices to soft robots. There are many hydrogel materials and hydrogel soft actuators that can be 3D printed and stimulated based on their respective stimulus e.g. thermal, electrical, pH, magnetic and light [84]. The common hydrogels include pluronic, alginate, chitosan, polyethylene oxide (PEO), polyethylene glycol (PEG), agarose and methylcellulose [85]. Recently, hydraulic actuation of hydrogels has been reported: most hydrogels consist of physically crosslinked dissipative polymer networks and covalently cross-linked stretchy polymer networks; physically cross-linked part of hydrogel was fabricated using a 3D printer by cross-linking the dissipative networks in pre-gel solutions [86]. A set of hydrogel actuators and robots composed of polyacrylamide (PAAm)-alginate hydrogels based on hydraulic actuation were fabricated.

### 3.4. Shape memory alloys

SMAs are the smart alloy materials that deform and regain their original shape when stimulated by heat and more common SMAs are (Cu–Zn, Cu–Zn–Al, Cu–Al–Ni, Ni–Ti, Ni–Ti–Fe, Fe–Pt) [87]. A 3D-printed soft robot has been presented based on SMA-based actuation; the body of the soft robot was printed by multi-material printable 3D printer using two materials: one is soft rubber-like material at room temperature, i.e. TangoBlackPlus polymerized by monomers containing urethane acrylate oligomer, Exo-1,7,7-trimethylbicyclo [2.2.1] hept-2-yl acrylate, methacrylate oligomer, polyurethane resin and photo initiator [88]. The other is rigid plastic at room temperature, i.e. Verowhite is a rigid plastic at room temperature polymerized with ink containing isobornyl acrylate, acrylic monomer, urethane acrylate, epoxy acrylate, acrylic monomer, acrylic oligomer and photo-initiators. For actuator installation, SMA (nitinol) wires were used in the form of coils to mimic the muscle as nitinol has the ability to deform when stimulated by heat. The SMA coils were tactfully embedded into the 3D-printed soft robot body and were electrically actuated. This soft body robot embedded with SMAs produced complex and robust gaits including inching and crawling.

In another work, a caterpillar-inspired soft-bodied rolling robot; GoQBot based on SMAs actuation has been presented in which the primary body was fabricated from positive and/or negative ABS plastic moulds built with a 3D printer using two kinds of castable commercially available silicone rubbers [64]. Similar to that of caterpillar muscles, the linear strains are converted into large displacements even due to small changes in temperature. The SMAs are actuated by resistive heating using pulses of the current that simulate muscle tetanus. To exploit the actuation of SMAs in 3D-printed soft robots, another study is being reported here; the authors of this study presented a tri-legged soft bot with spider mimicked [63]. A customized rotational multi-head 3D printer was used to make the structure of tri-legged soft bot using two materials: one is rigid (epoxy-based resin) and the other is flexible (polyurethane-based resin). Both the materials were cured instantly by UV lasers attached to the multi-head. The SMA actuator was embedded into the body of tri-legged bot during the 3D printing fabrication, SMA generated a pulling force by deforming its shape in form of contraction when an electric signal is applied. The authors have claimed that the 3D-printed soft robot powered by SMA actuator has a stable motion with a speed of 2.7 mm s^−1^.

### 3.5. Fluidic elastomers

FEAs are the relatively new class of smart materials having characteristics like low power actuation, highly extensible and adaptable. These elastomers consist of synthetic elastomer films that operate by the expansion of embedded channels under pressure and when pressure is applied, the FEAs will keep their position with little or no additional energy consumption; they can be powered hydraulically or pneumatically but powered pneumatically has advantage because it provides a low viscosity power transmission medium [89,90]. Using FEAs, a work has been reported which demonstrates a highly extensible sensing skin integrated with soft, pneumatic actuators (FEAs) using ‘DIW’ 3D printing technique [91]. The rheological properties of FEAs were tailored by preparing the homogeneous blend of silicones with high and low molecular weights. The shear thinning and yield stress characteristics of FEAs were adjusted to make them flowable and printable through nozzle of DIW system.

### 3.6. Smart soft composite

Smart soft composite (SSC) material based on a typical smart actuator: a SMA; an anisotropic material (ABS); and a polymer (PDMS) matrix has been fabricated by multi-nozzled 3D printer [92]. It showed a large in-plane/bending/twisting deformation with the help of SMA, ABS embedded in PDMS matrix. Silicone-based elastomers have been used for soft robots actuated by inflation of a pneumatic network as they have the capacity to bear the large strains (>700%) [48]. The composite structure of silicone elastomers (Ecoflex (Smooth-on, ebay, USA) and PDMS (4science.net Seoul, South Korea)) was also used to fabricate the soft tentacles as PDMS is less flexible then Ecoflex so used as rigid part of tentacle while Ecoflex was used for more flexible part [49]. Magnetorheological (MR), electrorheological (ER) and thermorheological (TR) fluids can be actuated by their respective magnetic/electric fields and temperature and have the potential to be used as soft robotics actuators. A soft mobile robot composed of multiple thermally activated joints driven by single actuator has been presented to describe TR activated soft robot [93]. This work described the locomotion of inchworm-like soft robot on flat smooth surfaces utilizing the active TR fluids to locally control the robot’s global response to external loading.

## 4. 3D-printed biological soft robots

**Figure 4.**
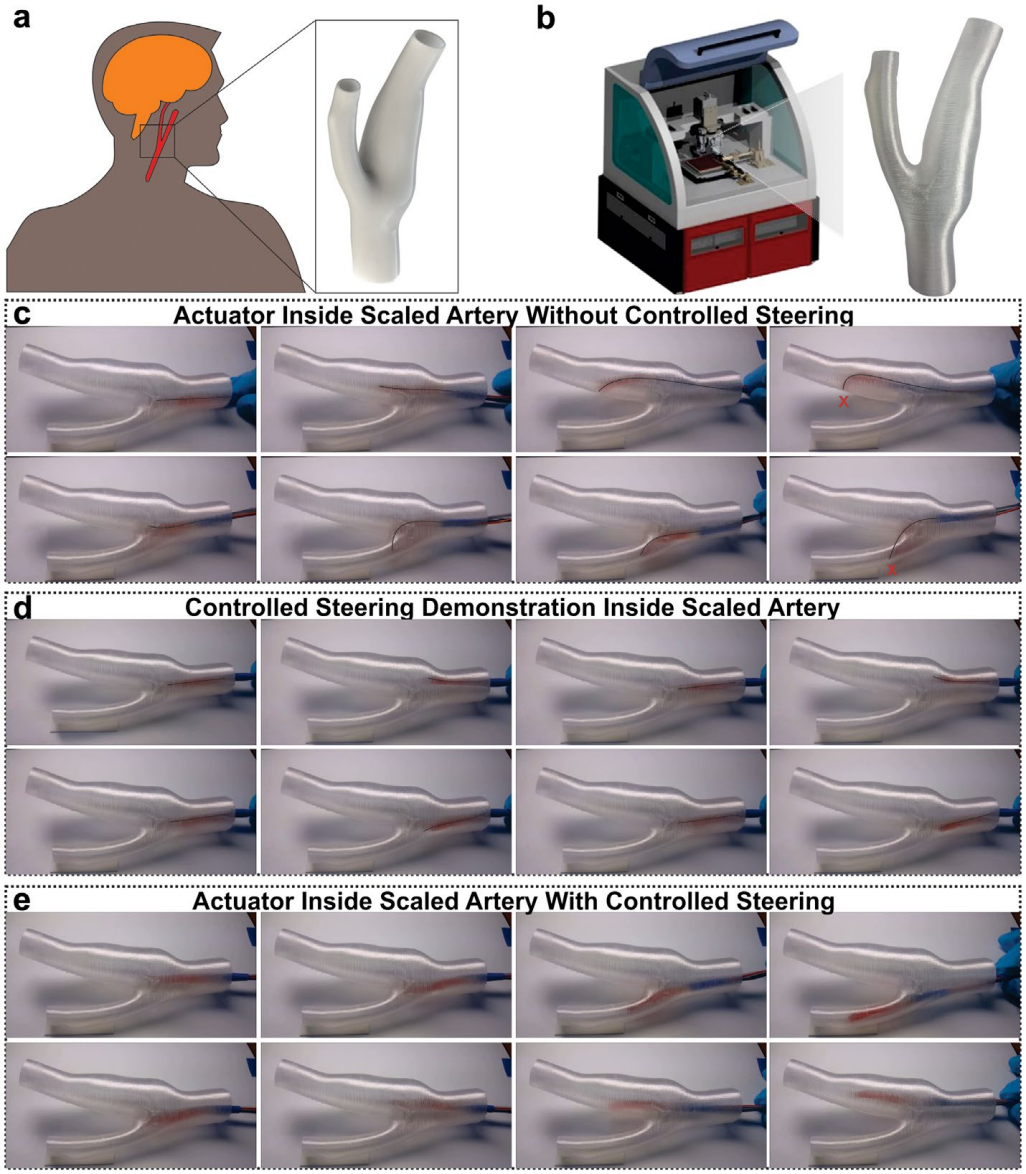
3D-printed bio-medical soft robot. (a) 3D CAD model of the carotid artery. (b) 3D-printed scaled version of the carotid artery. (c) Traveling of omnidirectional robot inside carotid artery without controlled steering. (d) Demonstration of static steering of omnidirectional robot inside carotid artery. (e) Traveling of omnidirectional robot inside carotid artery [4].

There is a vast variety in soft robots and actuators that are being developed to target the current biological issues. The robots can range from active implants to surgical and diagnosis robots depending on the target application. The two main categories in which the biological soft robots can be divided are *in vitro* robots (operating outside the body) and *in vivo* robots (operating inside a living organism). Just like the term soft robot refers to at least one major soft component of the robot, the term 3D printed refers to at least one component of the system fabricated through the help of additive manufacturing technology. 3D printing is usually used to only fabricate the soft robotic parts that are either not possible or are very difficult to fabricate through conventional techniques. In this section, we will specifically discuss the 3D-printed soft robots for *in vitro* and *in vivo* applications. Figure 4 shows the biological soft robot 3D printed using AMM’s multi-material 3D printing system.

### 4.1. 3D-printed in vitro robots

*In vitro* means anything that operates outside the body of a living organism in a controlled environment. 3D-printed soft robots have started to find their way in solving biological problems involving procedures carried outside the body of organisms. The edge soft robotics have over the rigid equipment is their flexibility, accuracy, degrees of freedom and ability to mould themselves according to the target body shape for applications in surgery, exoskeletal active implants, therapeutic systems, diagnosis, artificial skin and so on.

A soft robotic glove has been developed by scientists at Harvard [94] for combined assistance and at-home rehabilitation. The robotic glove comprises soft actuators with moulded elastomeric chambers to induce the specified motion through fluid pressurization. The glove is fabricated in multiple stages with the mould for the elastomers fabricated by 3D printing. The 3D printing enables to fabricate the soft robot’s size and shape exactly according to the patient’s requirement. The soft robotic glove can help in restoring movement for the disabled by providing specific physiotherapy.

Similar *in vitro* soft robots have been developed for active exoskeletal prosthetics and rehabilitation purpose using 3D printing fabrication techniques [95]. A soft robot for gait rehabilitation of spinalized rodents has been developed with 3D-printed main frame of the robot and the mould for soft actuators [50]. A soft robotic sensing unit for human gait measurement with printed soft sensors and electronics and 3D-printed mould for assembly has been developed [96]. 3D-printed flexible electronics for monitoring of vital signs have been developed to enable truly soft bio-robots [97]. 3D printing assisted fibre-reinforced soft actuators capable of following complex trajectories have been fabricated to develop active prosthetics for amputees [98]. Researchers from Australia have 3D-printed flexure hinges for soft monolithic prosthetic fingers [99] that can be combined with the soft actuators and 3D-printed framework to develop a complete working prosthetic hand. 3D-printed stretchable electronics including soft sensors and actuators are also being developed that can be combined with the exoskeletal implants to accurately mimic the bio-functionality of the actual human organs conferring the senses like touch and heat to the artificial robotic organs [58,79,100,101]. The soft electronic sheet is also referred to as the artificial skin by some researchers as it plays the role of actual skin on robotic organs [80,102–104]. Instrumented cardiac micro-physiological devices have been developed via multi-material 3D printing that is capable to replace the animal models for clinical drug testing. Most of the organ-on-chip models that are aimed to replace the animal study are 2D and are static models of the cultured cells of the organs to be tested. The active 3D-printed working model of the organs with embedded sensors provides a way of non-invasive testing of tissue contractile stresses inside cell incubator environment [105]. Other examples of soft muscular systems inside the human body include stomach, tongue and diaphragm. These 3D-printed real-life muscular soft robotic models fabricated using bio-compatible materials and actual cultured cells can revolutionize the organ-on-chip research and can soon replace the current animal study-based diagnosis and drug testing process [106–108].

3D-printed soft robotics is also paving its way for surgical applications to perform complex precise movements and gripping objects in a way that is not viable using rigid robotic tools [52,61]. The soft robotic grippers mimic the actual human fingers to perform their tasks, thus combining the agility and dexterity of a human with the precision of a computer [49,56,109–111].

### 4.2. 3D-printed in vivo robots

Anything operating inside the body of a living organism and performing its functions is known as an *in vivo* system. 3D-printed soft robots are being used for *in vivo* surgeries, organ implants, targeted drug delivery, diagnosis of various disease and conditions and also in the treatment of certain medical conditions. For *in vivo* applications, there is a huge potential for soft robotics as the internal body structure is too complicated to reach using rigid materials. Also, most of the internal body organs and tissues, except bones, are soft structures and require similar structures for their repair and replacement. 3D-printed soft and smart robotic implants called tracheobronchial splints have been developed to automatically adjust their shape and size inside the blood vessels to treat tracheobronchial collapse in tracheobronchomalacia [112]. The major advantage of this active implant is its personalization for every pediatric patient. A soft 4D bio-material is printed by 3D SLS printing using the 3D model based on patient-specific design. A number of active internal organ implants and repairs have been targeted using 3D-printed soft robots. A 3D-printed soft silicone heart-inspired pump has been fabricated that can one day enable artificial heart implants after further improvements [113]. 3D-printed soft bio-bots using actual cardiomyocytes and bio-compatible hydrogels have been fabricated that will one day repair the damaged heart tissues by the direct 3D printing of the soft actuators on to the subject’s heart [59,114]. A soft robotic sleeve mimicking the material properties and natural motion of the heart has been developed and tested *in vivo* on pigs [115]. The 3D-printed implant increases the ejection output in the hearts of pig cadavers. This device is attached conformal to the heart surface without causing any inflammation or injury. 3D-printed soft biological machines powered by actual muscles are the first step towards the repair of damaged muscles or replacement with fully functional 3D-printed muscles inside the body [116]. 3D-printed soft micro-bio-bots can be employed for *in vivo* monitoring of various conditions and disease. These robots can travel through blood vessels and food track and reach the inaccessible areas of the human body [117,118]. They can provide information of the vitals in the vicinity though embedded sensors and can also deliver targeted drug to the affected areas. Another form of 3D-printed soft robotic actuators can be used in endoscopic monitoring and surgery by easily steering them through the vessels owing to their soft form and multiple degrees of freedom in motion [119]. Table 2 summarizes the *in vitro*/*vivo* biological soft structures.

**Table 2.** 3D-printed in vitro and in vivo soft structures.

Category	Type	Function	Soft material	3D printing technology	Ref.
*In vitro*	Exoskeletal Implant	Rehabilitation	Neoprene	3D-Printed mould	[94]
	Wearable exoskeleton	Therapy and monitoring	Silicone rubber	3D-Printed mould	[96]
	Electronic soft skin	Physical senses in implants	Silicone	Direct ink writing	[100]
	Electronic soft skin	Physical senses in implants	ABS/PDMS/SMA	3D-Printed Scaffold	[104]
	Life-like organs on a chip	Drug discovery and testing	Real organs cultured tissues	Multi-material 3D printing	[105]
*In vivo*	Tracheobronchial implants	Treatment of bronchial disorder	PCL and Polyester	SLS printing	[112]
	Soft robotic sleeve for heart	Supports and strengthens functionality	Silicone	3D-printed alignment fixture	[115]
	Bio-morph cantilever	Organ repair	Cardiac cell sheet/hydrogel PEGDA	Direct 3D printing	[59]
	Muscle powered biological machines	Muscle repair or replacement	Cultured muscle cells, PEGDA	3D-printed mould and skeleton	[116]

## 5. Conclusions and future trends

3DP offers unique advantages with respect to fabrication soft robots with a complex external anatomy shape and internal porous structure. Coupling complicated porous 3D design with AM techniques can create a range of soft robots with bone and muscles from various materials. For soft robots, control over mechanical behaviour while retaining the designed structure is very important. The outside part of the soft robot is denser than the inner part, mimicking such structures is very difficult using 3DP, which is a challenge related to non-uniform shrinkage during sintering. Apart from the issues related to all 3DP settings as well as selecting suitable materials, usage of 3D printing in soft robotics is still a big challenge. In fact, there are many obstacles along this long and difficult road. The gap between the concept and the practical use of 3D printing comprises the main factors: the necessity of fabricating soft robots based on these design specifications. Despite all the advances in materials science, and system development there are still major gaps in this field relative to the printing multiple materials and adhesion between materials.

This field is still new and not much commercial fabrication has been done. A lot of 3DP techniques like SLS and ink jet printing soft robots have been successfully fabricated. Most reports have been limited to using models as guide templates and for *in vitro* and *in vivo* experiments, whereas implantations of 3D-printed soft robots in the human body are still rare. Demand for 3D printing technologies such as SLS and 3DP will increase in the future due to their capability to make custom soft robots that can be tailored for application-specific and defect-specific needs. Integrating all key points mentioned as well as finding solutions to cope with the challenges and issues are important in guiding the progress of these techniques towards achieving the objective of advanced soft robots. Lastly, commercial success depends on new innovation in soft lithography, 3D printing and other rapid prototyping technologies to mass produce soft structures and robots that are inexpensive and satisfy market demand.

## Disclosure statement

The authors declare that they have no conflict of interest.

## Funding

This work was supported by the National Research Council of Science & Technology (NST) grant by the Korea Government (MSIP) [grant number CRC-15-03-KIMM].
